# Subungual Hematoma

**DOI:** 10.7759/cureus.48952

**Published:** 2023-11-17

**Authors:** Anitej Akella, Anjali R Daniel, Murdoc B Gould, Rohan Mangal, Latha Ganti

**Affiliations:** 1 Biomedical Sciences, University of Central Florida College of Medicine, Orlando, USA; 2 Biology, Emory University, Atlanta, USA; 3 Chemistry, Rollins College, Orlando, USA; 4 Medicine, University of Miami Leonard M. Miller School of Medicine, Miami, USA; 5 Medical Sciences, The Warren Alpert Medical School of Brown University, Providence, USA; 6 Emergency Medicine and Neurology, University of Central Florida College of Medicine, Orlando, USA

**Keywords:** nail bed trauma, subungual hematoma, distal phalanx fracture, foot trauma, trephination

## Abstract

The authors present the case of a 64-year-old male who presented to the emergency department due to foot trauma. He sustained a large subungual hematoma, which was drained. Following the procedure, the patient achieved complete resolution of his pain. He also reported no complications at two-week phone follow-up. The management of subungual hematoma, including the trephination procedure, is discussed. Potential complications, although rare, are reviewed.

## Introduction

Subungual hematoma, a common nail injury, is characterized by the collection of blood (hematoma) underneath a toenail or fingernail. It typically occurs due to a direct trauma to the nail, and its prevalence is notable in both general and specific populations, such as athletes. The exact prevalence of subungual hematoma is difficult to ascertain due to its frequent occurrence in minor injuries and the likelihood of underreporting. However, it is a common presentation in emergency departments, especially among individuals engaged in physical activities or manual labor. Subungual hematomas are seen across all age groups but are more prevalent in adults due to occupational and recreational activities. Athletes, particularly those involved in sports like soccer, basketball, and running, are at a higher risk due to repetitive trauma to the toes [1]. They are also more common in warmer climates, where people tend to go barefoot or wear open-toed shoes more often. Ill-fitting shoes can also cause subungual hematomas due to repetitive trauma [2]. The primary risk factor for subungual hematoma is acute trauma to the nail. This trauma can be a result of incidents such as dropping a heavy object on the toe or finger, crushing injuries, or repetitive stress as seen in long-distance runners. Chronic repetitive trauma may also lead to subungual hematoma in certain occupations, such as construction workers or dancers.

In addition to external trauma, certain systemic conditions can predispose individuals to subungual hematoma. These include coagulopathy, anticoagulant therapy, and other conditions that impair blood clotting mechanisms. Patients with peripheral vascular diseases or diabetic neuropathy may have an altered pain response, leading to a delay in seeking treatment for nail trauma, thereby increasing the risk of complications.

The clinical presentation of a subungual hematoma is typically straightforward. The most common symptom is sudden, throbbing pain following nail trauma, which is due to the pressure of the blood collection under the nail plate. The affected nail appears discolored, ranging most commonly from maroon to black, but can also be blue-white [3]. The size of the hematoma can vary, and this has implications for management. Small hematomas (<25% of the nail area) may not require intervention, while larger hematomas may necessitate decompression for pain relief and to prevent complications such as nail dystrophy or secondary infection.

## Case presentation

**Figure 1:**
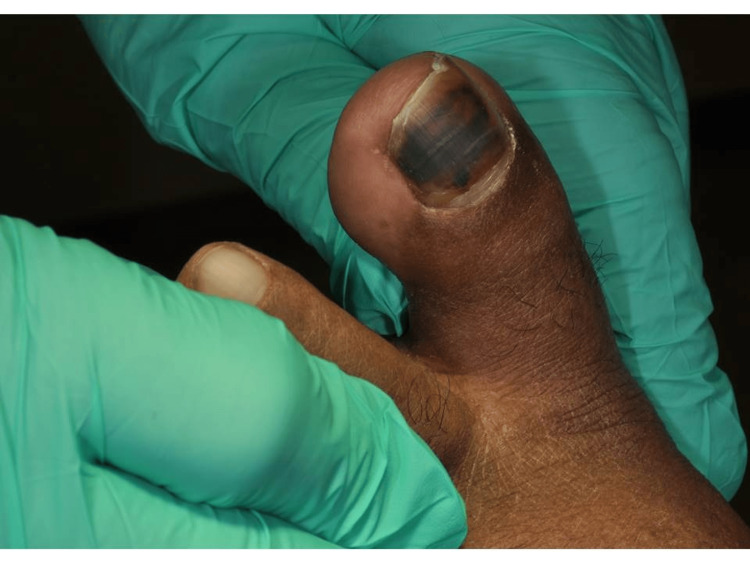
Clinical photograph of the patient demonstrating subungual hematoma

A 64-year-old male who accidentally dropped a stack of bricks on his great left toe presented to the emergency department. He was using the bricks to pave his driveway when they slipped from his hands. His past medical history was significant for coronary artery disease for which he was on a daily aspirin. His vital signs were as follows: blood pressure: 150/87 mmHg, temperature: 97.8°F, respirations: 18 breaths per minute, pulse: 82 beats per minute, and oxygen saturation: 97% on room air. He was ambulating without difficulty but had pain localized to his great toe. He did not have any other injuries. Plain radiography of his foot did not reveal any evidence of fracture or dislocation. There were no lacerations of the foot. His great toe demonstrated evidence of a subungual hematoma that encompassed approximately 70% of his toenail surface area (Figure 1).

He underwent a trephination for his traumatic subungual hematoma. Following sterile precautions, the skin surrounding the subungual hematoma was cleaned with a povidone-iodine solution. A sterile 14-gauge needle was used to make a hole in the nail, using a controlled twisting motion, and a light amount of pressure was applied onto the center of the hematoma until no more resistance was felt. Penetration was stopped thereafter, to avoid damage to the underlying nail bed. A small amount of pressure was applied around the tip of the toe and hematoma to aid with draining. The patient was discharged home with instructions to keep the trephination site clean and dry for one to two days. At two-week phone follow-up, the patient reported no complications.

## Discussion

Trephination is the typical treatment for subungual hematomas. In the majority of cases, patients have a good outcome after trephination, and further treatment is not necessary [4]. Trephination uses a hollow tip needle, electrocautery, or heated paper clip [5]. Traumatic subungual hematomas usually present with sharp-edged color changes to the nail plate [3]. In a trephination, a heated paper clip, needle (preferred), or electrocautery is used to penetrate the nail plate. The patient will experience dull pain because of the pressure exerted from the drilling or torquing motion; however, the small hole that was created will decrease the internal pressure in the nail and allow the blood to drain. Whether or not the nail was properly penetrated can be confirmed by testing for the emergence of dark blood from the hole onto the nail plate [6].

While often considered a minor injury, subungual hematoma can occasionally lead to several complications [6]. The disruption of the nail bed and the potential for open fractures increase the risk of bacterial infection. Infections can range from localized cellulitis to more severe cases like osteomyelitis, especially if the injury involves bone [7]. (Our patient did not have an underlying fracture.) The presence of a subungual hematoma can create an environment conducive to bacterial growth, particularly if the hematoma is not adequately drained or if there is an associated laceration. A study of 47 emergency department patients with subungual hematoma greater than one half of the size of the nail bed had a 60% incidence of a laceration requiring repair [8].

If not promptly drained, the persistent pressure from the accumulated blood can lead to permanent changes in the nail matrix, resulting in nail dystrophy. This may manifest as ridging, splitting, or a permanent change in nail shape. In severe cases, complete nail loss can occur. A subungual hematoma can sometimes mimic the appearance of subungual melanoma, leading to a misdiagnosis [9]. This is particularly challenging in chronic or recurrent cases where the typical history of trauma may be absent. A high index of suspicion and, if necessary, a biopsy can help differentiate between the two. In our case, there was a clear history of antecedent trauma, so this differential is less relevant.

While pain is an immediate consequence of the injury, inadequate management of a subungual hematoma can lead to prolonged discomfort. This is particularly true in cases where the pressure from the hematoma is not relieved, leading to ongoing pain and sensitivity. Separation of the nail from the nail bed, known as onycholysis, can also occur following a subungual hematoma [8]. This can lead to secondary infections or may cause the nail to catch on objects, exacerbating the injury. In patients with underlying conditions such as diabetes or peripheral vascular disease, healing can be significantly delayed. These patients are at a higher risk of complications, and their management should be tailored accordingly. And finally, in rare cases, particularly with severe crush injuries, a subungual hematoma can contribute to compartment syndrome in the digits. This is a surgical emergency requiring prompt intervention to prevent ischemic damage to the digit.

## Conclusions

This case reviews the presentation of a typical subungual hematoma after which the patient did not experience any complications. While usually a minor injury that is easily treated with drainage of the hematoma, complications can arise, particularly in patients with underlying systemic conditions or when associated with more significant trauma. Therefore, a thorough clinical assessment and an individualized approach to management are essential for optimal patient outcomes.

## References

[1] James V, Heng TY, Yap QV, Ganapathy S (2022). Epidemiology and outcome of nailbed injuries managed in children's emergency department: a 10-year single-center experience. Pediatr Emerg Care.

[2] (2023). Subungual Hematoma. https://www.aocd.org/page/SubungualHematoma.

[3] Metin MS, Elmas ÖF (2020). Dermoscopic diagnosis of subungual hematoma: new observations. Postepy Dermatol Alergol.

[4] Meek S, White M (1998). Subungual haematomas: is simple trephining enough?. J Accid Emerg Med.

[5] Blereau C, Radloff S, Grisham J (2022). Up in flames: the safety of electrocautery trephination of subungual hematomas with acrylic nails. West J Emerg Med.

[6] Patel PS, Ganti L (2022). Subungual hematoma drainage. Atlas of Emergency Medicine Procedures.

[7] Grisafi PJ, Lombardi CM, Sciarrino AL, Rainer GF, Buffone WF (1989). Three select subungual pathologies: subungual exostosis, subungual osteochondroma, and subungual hematoma. Clin Podiatr Med Surg.

[8] Simon RR, Wolgin M (1987). Subungual hematoma: association with occult laceration requiring repair. Am J Emerg Med.

[9] Deinlein T, Hofmann-Wellenhof R, Zalaudek I (2016). Acral melanoma mimicking subungual hematoma. J Am Acad Dermatol.

